# Fostering of a wild, injured, juvenile by a neighbouring group: implications for rehabilitation and release of Barbary macaques confiscated from illegal trade

**DOI:** 10.1007/s10329-019-00729-w

**Published:** 2019-06-03

**Authors:** Liz A. D. Campbell

**Affiliations:** 1 International Fund for Animal Welfare (IFAW), Azrou, Morocco; 2 WildCRU, Department of Zoology, University of Oxford, Oxford, UK

**Keywords:** Wildlife rehabilitation, Illegal wildlife trade, Reintroduction, Primate adoption, Consolation, Third-party affiliation

## Abstract

Illegal live trade is one of the greatest threats to endangered Barbary macaques. Since trade focuses on young and Barbary macaques show extensive alloparental care, rehabilitation and release into wild foster groups is a promising strategy to free space in sanctuaries to allow continued confiscations, improve individual welfare, minimize captive care costs, and reinforce wild populations. However, little documentation exists on Barbary macaque releases and the age at which released macaques would be accepted by foster groups is unknown. Here, I report on a wild, nearly 3-year-old Barbary macaque male that was separated from his group after serious injury from a car collision. A neighbouring group found the distressed juvenile 2 days later, approached, groomed, and affiliated with him, and accepted him into the group. He remained with the foster group for 4 months until returning to his natal group. This observation challenges concerns that only infants and young juveniles may be accepted into non-natal groups, suggesting that even older confiscated juveniles could be candidates for release. This also adds to the literature on behavioural responses to distressed and injured individuals, demonstrating the capacity of wild monkeys to exhibit affiliative behaviours even towards unfamiliar distressed conspecifics, providing a rare example of potential consolation of an unknown individual. As so little documentation currently exists on the acceptance of Barbary macaques into wild foster groups, this observation provides valuable information for developing rehabilitation and release strategies for confiscated individuals, which can be critical for continuing to fight illegal trade of this endangered species.

**Electronic supplementary material:**

The online version of this article (10.1007/s10329-019-00729-w) contains supplementary material, which is available to authorized users.

## Introduction

The Barbary macaque (*Macaca sylvanus*) is an endangered primate found only in Morocco and Algeria, with an estimated population of 10,000 left in the wild (Fooden 2007). The species has faced considerable population declines, estimated at 50% over the past three generations, with further declines predicted (Butynski et al. 2008). The primary cause of recent declines and greatest immediate threat to their survival is live illegal trade (van Lavieren 2008). Young macaques are poached from the wild to be used primarily as pets in the international market, as well as photo props and pets in Morocco and Algeria (van Lavieren 2008). Annual harvest rates each year between 1995 and 2009 were conservatively estimated to be 150% what the population could sustain (van Lavieren 2008) and Barbary macaques are the most seized mammal in the European Union listed on the Convention on International Trade in Endangered Species of Wild Fauna and Flora (CITES), accounting for nearly a quarter of all live mammal seizures from 2001 to 2010 (van Uhm 2016). In response, the Barbary macaque was recently uplisted from CITES Appendix II to Appendix I (CITES 2016) and stricter national laws for its protection were enacted (Moroccan Law no. 29-05). The high numbers of macaques surrendered or confiscated from trade have caused many sanctuaries and zoos to reach capacity. For example, French police reported in 2007 that they seize approximately 50 macaques each year (van Lavieren 2008). Long waiting lists to accept macaques can result in many animals being euthanized and a lack of placement options can reduce motivation to confiscate smuggled or illegally held macaques, thus impeding the fight against the illegal trade (van Lavieren 2008). As a result of limited space in long-term captive care facilities, high costs of care for captive animals, welfare concerns of animals in captivity, and the endangered conservation status of the species, one of the most desirable options for Barbary macaques rescued from illegal trade is rehabilitation and release to the wild, when appropriate.

Barbary macaques live in highly social multi-male multi-female groups and social bonding is important for their survival in the wild (McFarland and Majolo 2013; Lehmann et al. 2015; Campbell et al. 2018a). Therefore, reintroduction to the wild should either be as groups formed in captivity or as individuals released into existing groups, rather than lone individuals. This species exhibits extensive alloparental care; individuals of all age-sex categories care for young (Small 1990; Paul and Kuester 1996), but particularly pronounced is the quality and quantity of care provided by adult males. Males exhibit all parental behaviours towards infants and juveniles except lactation, including prolonged carrying, protection, providing support in agonistic conflicts, grooming and playing (MacRoberts 1970), and thermoregulatory huddling (Campbell et al. unpublished; see Campbell et al. 2018a). Males exhibit preferences for particular infants and juveniles (MacRoberts 1970; Kuester and Paul 1986), though not as a result of paternity, potential paternity, or relatedness to the mother (Paul et al. 1992, 1996; Ménard et al. 2001). Because illegal trade focuses on young macaques, the extreme alloparental care characteristic of this species can be useful for reintroduction of rescued macaques to the wild, as non-natal groups can adopt young macaques (Waters et al. 2016) and males in particular can act as foster parents (MacRoberts 1970; Waters et al. 2016). However, decisions regarding rehabilitation and release are impeded by the lack of data on Barbary macaque releases and adoptions (see Beck 2017). It is thus currently unknown which factors affect whether an individual would be accepted into wild foster groups, particularly the age at which juveniles may be accepted.

Infant handling is highly seasonal in Barbary macaques, with greatest interest towards infants under 6 months old and declining with age (Small 1990; Paul and Kuester 1996; Waters et al. 2016). This caused concerns that perhaps only very young macaques should be considered potential candidates for release into foster groups, for fear that older juveniles may not be accepted [personal communication with the International Fund for Animal Welfare (IFAW) and Le Haut Commissariat aux Eaux et Forêts et à la Lutte Contre la Désertification (HCEFLCD)]. However, no data on this currently exist and documentation on Barbary macaque adoptions and releases is limited: Paul and Kuester (1996) reported the successful adoption of an unweaned infant by a lactating, unrelated female in semi-captive Barbary macaques after being kidnapped from its mother by another female and Waters et al. (2016) described releases of three confiscated infants and one juvenile. Two infants, approximately 6 months old, were released near a wild group in Ifrane National Park, Morocco; one fled at release and was not seen again and the other was adopted by an adult male. An 8-month-old male released near a wild group was carried away by adult males, and a released 1.5-year-old female joined a group vocalizing nearby (Waters et al. 2016). In addition, MacRoberts (1970) described a presumably orphaned 2-year-old male in Gibraltar who was returned to his group following temporary absence and adopted by an adult male, who extensively cared for and protected him. Thus, although there are good indications that young Barbary macaques could be successfully fostered by wild groups based on the limited documentation in this species (Waters et al. 2016) and successes in other primates (reviewed in Beck 2017), many questions remain. Principal questions for developing rehabilitation and release strategies include the appropriate age at which macaques may be considered candidates for release into foster groups and whether only young macaques are likely to be accepted is indeed the case.

Here, I report on the acceptance of a wild, nearly 3-year-old Barbary macaque male into a neighbouring group after serious injury caused him to become separated from his natal group. This observation challenges concerns that only infants and young juveniles may be accepted into wild foster groups and adds to the literature on primates’ behavioural responses to others’ injury and distress, showing that wild monkeys can provide affiliative and potentially consolatory behaviours even towards unfamiliar extra-group individuals.

## Study site, subjects and methods

Fifteen groups of Barbary macaques in Ifrane National Park, Morocco, are monitored regularly as part of ongoing conservation and research efforts by IFAW, in partnership with the Moroccan government (HCEFLCD), initiated in 2015 by the Moroccan Primate Conservation Foundation. The natal group (Blue Group) was first identified and studied in 2013 (Waterman 2017; Waterman et al. in revision). The foster group (MonkeyWatch Group) was first identified and studied in 2014 by the author as part of an ecotourism program established by the Moroccan Primate Conservation Foundation. Both groups have been continuously monitored since. All adults, subadults, and some juveniles in both groups are identifiable by the observers (author LADC and IFAW Community Scout AH) by visual characteristics. At the time of the observation, Blue Group consisted of 7 adult females (5+ years old), 6 adult males (6+ years old), 6 infants (< 1 year old), and 12 immatures (juveniles/subadults) and MonkeyWatch Group consisted of 6 adult females, 4 adult males, 5 infants, and 19 immatures.

The observation occurred along national road N13 in Ifrane National Park (N 33.415522, W − 5.179324), a region of overlap in the home ranges of these two groups and others (Campbell, unpublished data; see Campbell et al. 2018b). Intergroup encounters at this study site range from immediate withdrawal by one group to lengthy, sometimes aggressive, contests; no affiliation between groups has been observed. The juvenile that is the focus of this observation was a 33-month-old male named Pipo, the son of a low-ranking female (PEN) (Campbell et al. 2018a). Photos and video were recorded with Sony DSC-HX50V cameras.

## Observation

**Fig. 1:**
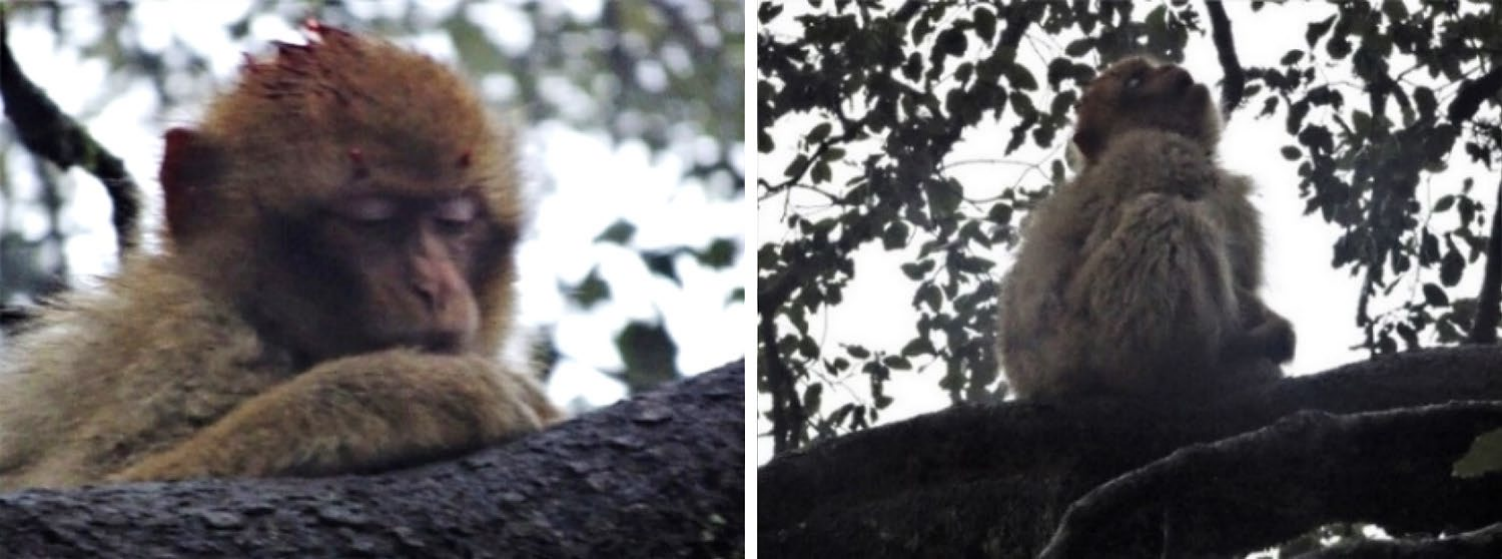
Injured juvenile Pipo retreated to a tree after being struck by a car and was left behind when unable to follow his natal group when they moved from the area (17:24 and 17:27, 20/03/2018)

The juvenile male, Pipo, was hit by a car on 20 March 2018 at 16:20. He suffered a head injury but was still alive. When he was hit, members of his group showed distress behaviours, i.e. screaming, and protective behaviours, acting aggressively towards observers that tried to approach him. Pipo retreated into a tree 8 m from where he had been hit. Several group members displayed affiliation towards him and a juvenile sat with him and groomed him as he appeared to be losing consciousness. At approximately 17:35 (1 h before sunset), his group left for their sleeping trees but Pipo was left behind in the tree (Fig. 1).

**Fig. 2:**
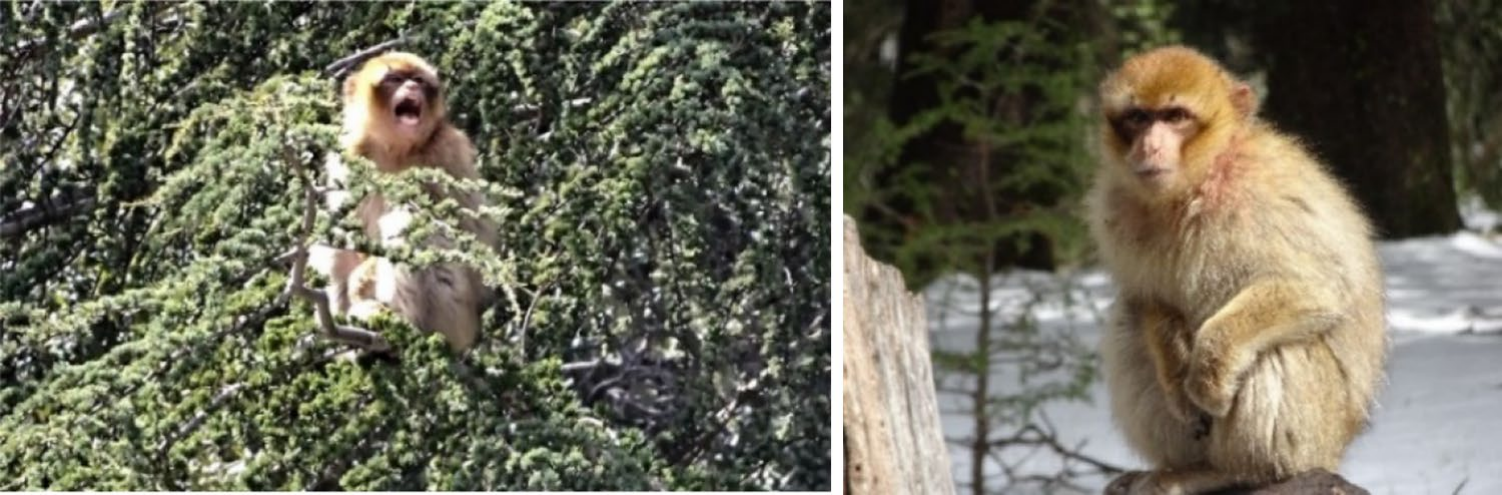
Injured juvenile Pipo **a** screaming alone in a tree at 11:33 (screenshot from Video provided as Online Resource) and **b** alone on the ground at 14:04 (22/03/2018)

**Fig. 3:**
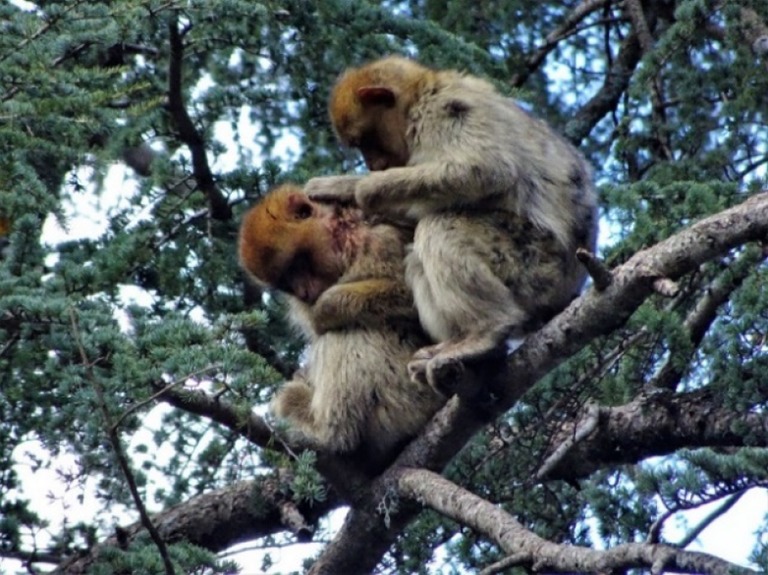
A juvenile from a neighbouring group inspects Pipo’s injuries and grooms him (14:46, 22/03/2018)

The following day Pipo was not found and it was suspected that he had died, but on 22 March he was found in the same tree, alone and screaming repeatedly (Fig. 2, see Online Resource video). He later left the tree to feed on the ground but continued screaming intermittently, then returned to the trees. At 14:40, a neighbouring group (MonkeyWatch Group) arrived. A juvenile approached Pipo, inspected his injuries, and groomed him (Fig. 3). Approximately 20 min later, an adult male also approached and groomed Pipo. When MonkeyWatch Group left the area later in the day, Pipo left with them. He did not scream again after MonkeyWatch Group arrived.

Pipo remained with the foster group even after he appeared fully healed from the car accident. He seemed to be well socially integrated in the foster group and was often seen grooming, playing and socializing with others, particularly with the adult male that had first groomed him, a subadult male, an adult female, and other juveniles. He remained with the foster group for 4 months until returning to his natal group at the beginning of July. The transfer to his natal group was not witnessed. Pipo is well and remains with his natal group at the time of writing (April 2019).

## Discussion

The acceptance of a nearly 3-year-old juvenile by a wild, non-natal group has important implications for rehabilitation and release of Barbary macaques rescued from illegal trade, challenging suggestions that only infants and young juveniles may be suitable for release into wild foster groups for fear that older juveniles may not be accepted. As a result of the high numbers of Barbary macaques in captivity that were confiscated or surrendered from trade, rehabilitation and release of suitable macaques (following quarantine and assessment of health, genetic and behavioural risks, coupled with long-term post-release monitoring) is desirable as it offers a potential solution to avoid sanctuaries from reaching capacity and thus allow continued confiscations, while also reducing long-term care costs associated with confiscations, improving welfare of rescued macaques, and reinforcing wild populations. Although most poached Barbary macaques are 1 year old or younger (van Lavieren 2008), macaques as old as 4 have been observed for sale in the illegal market (Bergin et al. 2018). The oldest Barbary macaque released into a wild group was 1.5 years old (Waters et al. 2016). Although juveniles continue to receive alloparental care (MacRoberts 1970), the decreasing attention towards young with age (Small 1990; Paul and Kuester 1996) and decline in care of an adopted infant by a wild foster male over time (Waters et al. 2016) caused concerns that foster groups may not accept older juveniles. The present observation shows that even older juveniles can be accepted by non-natal groups, suggesting that age alone should not disqualify juveniles as potential release candidates. However, appropriate assessment of suitability must first be conducted, as older rescued macaques have likely been in captivity longer, rather than poached at that age, and thus may be more likely to have developed abnormal behaviours or lack critical skills for living in the wild. Release of older juveniles may have several advantages, as dehydration and malnutrition of infants is a concern if foster parents do not lactate (Hrdy 1976; Thierry and Anderson 1986; Waters et al. 2016) and older wild orphans are more likely to survive (Thierry and Anderson 1986; Boesch et al. 2010). Infanticide risk may be an important consideration for release age in some species, but macaque social organization does not predict male infanticide (Yamada and Nakamichi 2006) and this is not a major risk in Barbary macaques.

Male Barbary macaques disperse (Mehlman 1986) and form strong social relationships with unrelated individuals (Young et al. 2014; Campbell et al. 2018a), so sex possibly contributed to Pipo’s acceptance. However, this case contrasts with dispersal in several ways: Pipo was much younger (< 3 years) than any documented dispersals in wild Barbary macaques (all > 5 years), the timing in March is inconsistent with Barbary macaque dispersals, which occur almost exclusively during the October–January mating season, and lone, young dispersing males often receive aggression from resident males, remain peripheral to the group, and may be unsuccessful at immigrating; thus young males often disperse in cohorts, whereas older males succeed through sexual associations with females (Paul and Kuester 1985, Mehlman 1986, Kuester and Paul 1999; personal observations 2014–2016). Pipo was approached by the foster group rather than the reverse, no aggression was displayed, and a juvenile and male first approached him, rather than females. Therefore, it seems that the foster group did not treat this event as dispersal. Adults continue to exhibit caretaking and protection towards juveniles of Pipo’s age (MacRoberts 1970), so age and circumstance likely contributed to his acceptance, rather than sex. Thus far, sex does not appear to affect acceptance of young Barbary macaques into foster groups; except for one female infant that fled upon release and was not seen again, all released macaques were accepted [one female infant, one male infant, one female juvenile (Waters et al. 2016), plus this male juvenile].

As in other observations of Barbary macaque adoptions (MacRoberts 1970; Waters et al. 2016), an adult male seemed to form the strongest relationship with Pipo, though he also often groomed, played, and socialized with a subadult male, adult female, and other juveniles. A juvenile also seemed to play an important role in Pipo’s integration, being the first to approach and potentially comfort him. Juveniles, though often overlooked, can play important roles in primate social networks (Fedurek and Lehmann 2017). Pipo’s social interactions contrast with those of an infant released into a wild group in this population (Waters et al. 2016) who failed to exhibit normal social behaviours, rarely played, never initiated play, and rarely socialized with individuals other than her male caretaker. Pipo played, groomed, and socialized with others, suggesting that the lack of social skills in the previous case may have been a consequence of her time in captivity and lack of socialization during a critical developmental period, rather than being in a new group.

Adoptions are not uncommon in wild primates (Hrdy 1976; Thierry and Anderson 1986), though it is usually by relatives or group members. However, a few intergroup (Agoramoorthy and Rudran 1992; Dunham and Opere 2016) and even cross-species (Izar et al. 2006) adoptions have been observed in wild primates. Although adaptive explanations for adoption have been proposed (e.g. adopting orphaned relatives or learning parenting skills, see Thierry and Anderson 1986), it is often maladaptive: adopting can increase females’ interbirth interval (Agoramoorthy and Rudran 1992; Cäsar and Young 2008) and compete with biological offspring (Dunham and Opere 2016) and in Barbary macaques, “kidnapping” by relatives can kill infants by starvation and dehydration (Kuester and Paul 1986). It therefore seems most likely that adoptions, group fostering, and kidnapping are non-adaptive by-products of alloparental care (Hrdy 1976; Thierry and Anderson 1986), which may be under strong positive selection (reviewed by Hrdy 1976; Dunayer and Berman 2018).

The various behavioural responses to the seriously injured juvenile, including distress, protection, exploration, and affiliation, have been previously described in wild Barbary macaques towards dying and dead groupmates (Campbell et al. 2016). Similar responses are reported in other primates (e.g. Biro et al. 2010; Anderson et al. 2010; Bezerra et al. 2014; Yang et al. 2016). This is, however, the first report of affiliation towards an unfamiliar, injured individual in wild Barbary macaques. There is growing evidence that various species can perceive others’ distress and provide appropriate responses, such as affiliation, to alleviate it (de Waal and Preston 2017). In a wide variety of species, uninvolved bystanders spontaneously approach victims of aggression to offer affiliation (e.g. Cozzi et al. 2010; Ikkatai et al. 2016; de Waal and Preston 2017), termed consolation when it serves to alleviate the victim’s distress (de Waal and van Roosmalen 1979). By this definition, true consolation has thus far been demonstrated in only a few species (apes, Tonkean macaques [*Macaca tonkean*], rodents, and possibly elephants [see de Waal and Preston 2017] and geladas [*Theropithecus gelada*; Palagi et al. 2018]), though many studies did not measure whether post-conflict third-party affiliation alleviated distress, rather than not finding evidence of it (e.g. Cools et al. 2008; Cozzi et al. 2010; Fraser and Bugnyar 2010). In Barbary macaques, consolation was partially supported as a function of post-conflict third-party affiliation: it reduced victims’ anxiety, but only when solicited by the victim (McFarland and Majolo 2012). Although consolation is thought to require recognition of others’ distress, it does not necessarily require complex cognitive perspective-taking (de Waal and Preston 2017). Emotional contagion, found in many species, may be an underlying mechanism, allowing distress recognition and motivating consolatory behaviour (Burkett et al. 2016; de Waal and Preston 2017; Carrillo et al. 2019).

The affiliative responses by individuals in MonkeyWatch Group could have alternative explanations, other than consolation. Affiliation may have served to avoid an aversive stimulus, i.e. stopping Pipo’s screaming. However, if that were the case, not approaching the screaming juvenile or leaving the area seem more likely responses. It may have been driven by personal distress, a self-motivated response whereby another’s distress evokes a matched state that the bystander seeks to alleviate through affiliation (de Waal 2008). However, if principally comforting themselves, individuals from MonkeyWatch Group would more likely affiliate with their own group, as close social partners are more effective at social buffering and stress relief (Young et al. 2014). Thus, the responses to Pipo seem to have been other-oriented, rather than self-oriented comfort-seeking. Finally, the affiliation could have been irrespective of his distress (Puga-Gonzalez et al. 2014). However, affiliation has never been observed during dispersal attempts or intergroup encounters (personal observations 2014–2016) and affiliation was observed in other cases of seriously injured Barbary macaques (Campbell et al. 2016). Unlike in post-conflict contexts, individuals from the foster group did not witness the stressful event and alternative hypotheses for affiliating with victims (protecting bystanders or victims from aggression, restoring social cohesion, or substituting for reconciliation, e.g. see McFarland and Majolo 2012; Palagi et al. 2018) do not apply, nor are they supported as functions of post-conflict third-party affiliation in Barbary macaques (McFarland and Majolo 2012). Greater distress can be more likely to elicit consolatory responses (Fraser and Bugnyar 2010; Palagi et al. 2014). Thus, such responses may be more likely when distress or injury is more evident, as with Pipo’s screaming and visible blood (Figs. 1, 2 and 3). The degree of observable injury was previously suggested to contribute to variations in Barbary macaques’ responses to injured and dead groupmates (Campbell et al. 2016). Juveniles were the first to approach and affiliate with Pipo, both from his own group immediately after injury and from the foster group. In bonobos and gorillas, immature individuals were most likely to console (Cordoni et al. 2006; Clay and de Waal 2013) and de Waal and Aureli (1996) suggested that young rhesus macaques have greater “consolatory disposition” than adults.

Affiliation towards an injured, unfamiliar individual by members of a different group is of interest, as it is generally close social partners or kin that display epimeletic caretaking behaviours towards injured, ill, and dying individuals (e.g. Chapman and Chapman 1987; Herrera and Heymann 2004; Anderson et al. 2010) and consolation/third-party affiliation (e.g. McFarland and Majolo 2012; Clay and de Waal 2013; Palagi et al. 2014), and experiments have found that consolation is not provided to unfamiliar, distressed individuals (Burkett et al. 2016). Close social partners may be more responsive to signs of anxiety, most interested in calming the victim, and most effective in alleviating stress. Pipo’s age may have contributed to MonkeyWatch Group’s response, as rats avoid unfamiliar stressed adults but approach and interact with unfamiliar stressed juveniles (Rogers-Carter et al. 2018). Approaching an unfamiliar individual stressed by an unknown stimulus was potentially risky for the foster group. A previous report of affiliation towards injured Barbary macaques also noted risky behaviour, where several males travelled at dark to a dying female (Campbell et al. 2016). Affiliation towards an unfamiliar, injured individual despite possible risks and aversions attests to the prosocial nature of this species and shows that social bonding is not required for affiliative, potentially consolatory, behaviour in wild Barbary macaques.

Having suitable placement options for confiscated Barbary macaques is critical for fighting illegal trade of this endangered species. Rehabilitation and release into wild foster groups, following quarantine and assessment of health, genetic, and behavioural risks and coupled with long-term post-release monitoring, is a promising strategy to reduce numbers of macaques in sanctuaries and thus allow further confiscations, while improving welfare of rescued macaques, minimizing captive care costs, and reinforcing wild populations. This observation of acceptance of a nearly 3-year-old juvenile into a non-natal group suggests that age alone should not disqualify rescued juveniles as potential candidates for release and demonstrates the capacity of wild monkeys to display affiliative, potentially consolatory, behaviour towards injured, distressed conspecifics, even extra-group individuals with whom they do not have a social relationship.

## Electronic supplementary material

Below is the link to the electronic supplementary material.

Supplementary material 1 (WMV 24513 kb)
